# Health-related quality of life assessment among patients with oesophageal cancer at Tikur Anbessa Specialised Tertiary Hospital in Ethiopia: a cross sectional study

**DOI:** 10.3332/ecancer.2024.1656

**Published:** 2024-01-18

**Authors:** Jilcha Diribi Feyisa, Adamu Addissie, Eva Johanna Kantelhardt, Girum Tessema Zingeta, Hiwot Saboksa Mideksa, Helen GebreLibanos, Tariku Mengesha, Mathewos Assefa

**Affiliations:** 1 Department of Oncology, Saint Paul’s Hospital Millennium Medical College, Addis Ababa 1271, Ethiopia; 2 Department of Radiation Oncology and Applied Sciences, Dartmouth Cancer Center, Lebanon, NH 03756, USA; 3 Global Health Working Group, Institute for Medical Epidemiology, Biometrics, and Informatics, Martin-Luther-University Halle-Wittenberg, Halle (Saale) 53170, Germany; 4 Department of Preventive Medicine, School of Public Health, Addis Ababa University, Addis Ababa 9086, Ethiopia; 5 Institute of Medical Epidemiology, Biometrics and Informatics, Martin-Luther-University Halle-Wittenberg, Halle (Saale) 53170, Germany; 6 Department of Oncology, School of Medicine, Addis Ababa University, Addis Ababa 9086, Ethiopia; 7 Department of Epidemiology, School of Public Health, Debre Birhan University, Debre Birhan 445, Ethiopia; 8 Department of Research, Saint Peter’s Specialised Hospital, Addis Ababa 21494, Ethiopia; a https://orcid.org/0000-0002-5344-5340; e https://orcid.org/0000-0003-0272-8460

**Keywords:** health related quality of life, oesophageal cancer, Ethiopia

## Abstract

**Background:**

In low-income countries, oesophageal cancer often presents at an advanced stage, leaving patients with limited curative treatment options. Furthermore, palliative treatments such as oesophageal stents or brachytherapy are lacking. This has a detrimental effect on their quality of life. In this study, we investigated the health-related quality of life of patients with oesophageal cancer at a tertiary hospital in Ethiopia.

**Methods:**

This cross-sectional study was conducted at Tikur Anbessa Specialised Tertiary Hospital in Ethiopia. The validated Amharic version of the questionnaire of the European Organisation for Research and Treatment of Cancer Core Quality of Life Questionnaire Cancer 30 (EORTC QLQ C-30) and the oesophageal cancer disease-specific questionnaire QLQ-OES18 were used to assess the quality of life of each patient.

**Results:**

The overall mean score for the EORTC QLQ C-30 was 35.43 (SD 18.04). The mean scores for the functional scales revealed that cognitive function was the highest, whereas role function was the lowest. The symptom scale results showed the highest score for pain and the lowest for diarrhoea. Dysphagia, choking, role functioning and financial difficulty correlated with the quality of life of patients with oesophageal cancer.

**Conclusion:**

Dysphagia, choking, role functioning and financial difficulty are important factors that affect the quality of life of patients with oesophageal cancer patients. Increasing the availability of palliative treatments for dysphagia to improve the quality of life in patients with oesophageal cancer is recommended.

## Background

There is no single definition for health-related quality of life (HRQoL). According to European Organisation for Research and Treatment of Cancer (EORTC), in general, HRQoL encompasses patients' subjective assessments of positive and negative elements of their cancer symptoms, including physical, emotional, social and cognitive functions, as well as disease symptoms and treatment side effects [1]. HRQoL has been identified as an important indicator of treatment decisions and prognosis in patients with oesophageal cancer [2].

The quality-of-life questionnaire (QLQ) was developed to assess the HRQoL of individuals with particular medical conditions [3]. The QLQ-C30 and QLQ-OES18 are two commonly used questionnaires for assessing HRQoL in patients with oesophageal cancer [4, 5]. Both questionnaires are validated and reliable tools for assessing HRQoL in patients with oesophageal cancer [4, 5]. The QLQ-C30 and QLQ-OES18 information can then be used to develop treatment plans and monitor patient progress.

A review of the literature has revealed that the HRQoL of patients with oesophageal cancer is generally lower [6]. Various factors such as physical symptoms, psychosocial distress, social support, disease stage, treatment regimens and patient demographics have been identified as contributing to this decrease in HRQoL [6]. Studies conducted in various settings, using validated HRQoL questionnaires such as the QLQ-C30 and the QLQ-OES18 have highlighted the different factors that influence the QoL of oesophageal cancer patients [6, 7].

In Ethiopia, patients with oesophageal cancer often present at an advanced stage owing to a variety of factors, such as low health-seeking behaviours, limited access to hospitals and trained personnel, lack of early screening or diagnosis facilities, and low literacy levels [8]. All of these factors significantly impact the quality of health of patients with oesophageal cancer. However, quality of life assessment for oesophageal cancer in Ethiopia remains largely unexplored. In this study, we assessed the HRQoL of patients with oesophageal cancer in Ethiopia using the QLQ-C30 and QLQ-OES18 questionnaires.

## Methods

This cross sectional study was conducted at Tikur Anbessa Specialised Hospital (TASH). Before the recent opening of radiotherapy centres at Jimma University Hospital and Haramaya University, TASH was the only RT centre in Ethiopia for more than 20 years [9, 10]. The study was conducted on patients treated from 27 February 2018 to 28 February 2019.

The division of the EORTC has prepared a standard instrument to evaluate HRQoL in patients with cancer [11]. The EORTC QLQ-C30 is one of the most widely used instruments for measuring HRQoL in cancer patients and has been validated in numerous studies [4] (Supplemental Table 1). A questionnaire was used to assess the HRQoL of patients with various types of cancer, including oesophageal cancer. The EORTC QLQ C-30 consists of a multi-item scale and a single-item scale. It includes five functional scales, three symptom scales, a global health status/QoL scale, and six items. Every multi-item scale comprises a diverse set of items; no item is repeated on other scales. The QLQ-OES18 consists of 18 questions that assess physical, psychological, social and functional aspects [5] (Supplemental Table 1). It is based on patient-reported outcomes and has been developed to provide a reliable and valid measure of HRQoL in people with oesophageal cancer.

All participants were interviewed by trained oncology nurses using two questionnaires: the validated Amharic version of the EORTC QLQ C-30 and oesophageal cancer disease-specific questionnaire QLQ-OES18, to assess the HRQoL for each patient (Supplemental Table 2). The tool was validated in Amharic, with an internal consistency of Cronbach’s *α* value of 0.81 (Cronbach’s *α* value >0.7), except for cognitive functioning domain (Cronbach’s *α* value of 0.29) for the QLQ C-30 [12] and used in different studies [12, 13]. The Amharic version of the EORTC QLQ OES-18 version 3 was validated as part of the current study, with a Cronbach's alpha value of 0.836, demonstrating its high reliability for measuring the health-related HRQoL of patients with oesophageal cancer (Supplemental Table 3). Data abstraction tools were used to collect socio-demographic and clinical characteristic data from the medical charts.

Five trained oncology specialist nurses collected data from the interviews. Additionally, two trained clinical oncology resident physicians working in the TASH oncology unit performed the data abstraction. The principal investigator conducted a 2-day training session to equip the data collectors and one supervisor with the necessary knowledge for the study. The training covered the objectives of the study, the questionnaire content, informed consent and data confidentiality.

The scoring procedures for the EORTC QLQ-C30 and QLQ-OES18 questionnaires were conducted in accordance with the EORTC scoring manual [14]. For the EORTC QLQ-C30, a raw score was calculated by taking the average of the items in a scale or single-item measure, and then applying a linear transformation to standardise the raw score, so that the score ranged from 0 to 100. The range of values for items contributed to the range of raw scores. A higher response level is indicated by a higher score. Therefore, a high score on a functional scale denotes a high and healthy level of functioning, a high score on a global health status/HRQoL scale denotes a high and healthy HRQoL, and a high score on a symptom scale/item denotes a high and problematic level of symptomatology [14]. The principles for scoring these scales were the same in all cases: 1. Estimate the average of the items that contribute to the scale, which is the raw score. 2. A linear transformation was used to standardise the raw score such that scores ranged from 0 to 100; a higher score represented a higher (‘better’) level of functioning or a higher (‘worse’) level.

### Operational definitions

Physical function: confined to bed, needed help dressing, washing and eating when the score was 0 and able to do strenuous activities were possible when the score was 100 [5, 14, 15].

Role function: is completely unable to work at a job or do household jobs when the score is 0 and able to do work at home and at work.

Emotional function: felt intense, irritable, depressed and worried a lot when the score was 0 and didn’t feel intense, irritable and did not worry when the score was 100.

Social function: when score was zero, physical function and medical treatment significantly disrupted family and social activities; when the score was 100, physical function and medical treatment had no effect on family and social activities.

Fatigue: did not feel at all weak or tired and did not need to rest at all when the score was 0, and did feel very weak and tired and needed to rest a lot when the score is 100.

Cognitive function: had a lot of difficulty concentrating and remembering things when the score is 0, and there is no difficulty concentrating and remembering things when the score is 100.

Nausea and vomiting: did not feel nauseated and vomited when the score was 0 and felt nauseated and vomited when the score was 100.

Pain: severe pain that significantly interfered with daily activities when the score was 100, but neither pain nor discomfort interfered with daily activities when the score was 0.

Global health status (QOL) is the outcome of the study and was dichotomized into poor quality (affected) and good quality (unaffected) based on a review by Koller and Lorenz [16] with 50 cut-off points. The review stated that a cutoff point of 50 could indicate clinical impairment or enhancement when using the EORTC questionnaire. For the QLQ OES18 questionnaire, an overall score was obtained by adding the responses to each item and then dividing by the number of items answered [5].

After checking the data for completeness and consistency, they were entered into the Epi Data Manager version 4.6.0.0, and exported to SPSS version 25. The data were then analysed using descriptive statistics, such as the mean and SD, to obtain a summary of the quality-of-life scores for the entire sample. Independent variables, such as functional and symptom scales of general and specific questionnaires, were considered continuous variables. Bivariate correlation analyses were conducted to explore the relationship between quality-of-life scores and other variables, such as sociodemographic and clinical characteristics. Finally, multivariate binary logistic regression analysis was conducted to examine the predictive power of each independent variable on quality-of-life scores.

## Results

### Socio-demographic characteristics of oesophageal cancer patients

Among 210 oesophageal cancer patients, 11 questionnaires were incomplete, resulting in 94.7% response rate. The average age of the participants was 52 ± 13 years (87 males and 112 females). The majority of the patients (*n* = 167, 83.5%) were married, with 82 (41%) of respondents being farmers and house wives each (Table 1).

### Clinical and pathologic characteristics of oesophageal cancer patients

Of the 199 patients, comorbid illnesses were present in 13 (6.5%) of the patients, with retroviral infections, hypertension and diabetes mellitus being the most common (3.0%, 2.0% and 1.5%, respectively). Dysphagia was the most common presenting symptom (*n* = 198, 99.5%). The majority of patients (*n* = 118, 59.3%) had an ECOG performance status of I. Clinical staging revealed that 87 (43.7%) of the patients had stage IVA and stage IVB 70 (35.2%) (Table 1).

### HRQoL score for oesophageal cancer

#### Global health status (Functional and symptom scale cores of EORTC QLQ C-30)

The EORTC C-30 evaluation of global health status (QOL) revealed an overall mean score of 35.43 (SD = 18.04). The mean scores for the functional scales revealed that the highest score was for the cognitive function scale (62.28, SD 28.25) and the lowest score was for the role function scale (20.79, SD 25.82). The symptom scale results revealed the highest score of 82.23 (SD 20.70) for pain and the lowest score of 9.76 (SD 24.79) for diarrhoea. The mean score for financial difficulties was 90.90 (SD = 16.32) (Table 2).

#### Symptom scales score of EORTC QLQ OES-18

The patients reported an average score of 40.23 (SD = 24.53) for dysphagia, 41.75 (SD = 36.89) for trouble swallowing saliva, and 39.05 (SD = 36.18) for choking while swallowing. Eating difficulty (mean score = 71.04, SD = 21.93) had the highest score, followed by pain (mean score = 71.99, SD = 23.95), dry mouth (mean score = 54.71, SD = 36.90), reflux (mean score = 50.92, SD = 29.19), and trouble with coughing (mean score = 46.12, SD = 37.00 (Table 2).

### Bivariate association between HRQoL and independent variables

#### Functional and symptom scale of EORTC QLQ C-30

From bivariate logistic regression, only variables with *p* ≤ 0.2 were selected for the multi variable logistic regression. Accordingly, the results of the bivariate analysis between HRQoL and the independent variables from the Functional and Symptom scales of the EORTC QLQ C-30 showed that there was a statistically significant association between HRQoL and the independent variables. Physical, role and Cognitive Function scales were significantly associated with HRQoL, with a COR (95% CI) ranging from 1.004–1.030, 1.008–1.033 and 1.002–1.026, respectively, and *p*-values of 0.009, 0.001 and 0.023, respectively. Among the symptom scales/items, fatigue, pain, nausea and vomiting, dyspnoea, and financial difficulties were significantly associated with HRQoL, with COR (95% CI) ranging from 0.969–0.993, 0.966–0.994, 0.980–0.999, 0.976–0.994 and 0.958–0.993, respectively, and *p*-values of 0.002, 0.006, 0.025, 0.001 and 0.006, respectively. There was no significant association between HRQoL and emotional function, social function, insomnia, appetite loss, constipation and diarrhoea, with COR (95% CI) ranging from 0.992–1.015, 0.995–1.018, 0.983–1.001, 0.988–1.003, 0.988–1.003, and 0.975–1.005, respectively, and *p*-values of 0.541, 0.243, 0.069, 0.266, 0.264 and 0.182, respectively (Table 3).

#### Symptom scale of EORTC QLQ OES-18

The results of the bivariate association between the functional and symptom scales of the EORTC QLQ OES18 and HRQoL among patients with oesophageal cancer showed that trouble coughing (COR 0.982–0.999, *p*-value 0.029) and trouble talking (COR 0.980–0.999, *p*-value 0.029) had the strongest association with HRQoL. There was also a moderate association between dysphagia (COR 0.974–1.00, *p*-value 0.057) and eating difficulty (COR 0.976–1.004, *p*-value 0.145). There was no significant association between HRQoL and the other variables included in the table, such as trouble swallowing saliva (COR 0.988–1.005, *p*-value 0.463), chocking (COR 0.985–1.003, *p*-value 0.163), dry mouth (COR 0.986–1.002, *p*-value 0.158), trouble tasting (COR 0.988–1.005, *p*-value 0.433), reflux (COR 0.987–1.008, *p*-value 0.645) and pain (COR 0.981–1.006, *p*-value 0.310) (Table 3).

#### Multi variable logistic regression of oesophageal cancer

The final model was constructed using backward stepwise logistic regression. A total of 18 independent variables were candidates for the multivariable logistic regression. The final model of oesophageal cancer was established by 187 participants, the remaining 12 were not analysed due to missing data. The final model was significant (*X*^2^_(4)_, 26.357, *p* < 0.01) correctly classifying good HRQoL by 72.7%. The model was a good fit, as shown by Hosmer-Lemeshow goodness-of-fit (*X*^2^_(8)_, 4.35, *p* = 0.824) and the model variation was explained between 13.1% (Cox and Snell R square) and 18.9% (Nagelkere R square).

HRQoL is affected by dysphagia, chocking, role functioning and financial difficulty. As there was a unit increase in dysphagia, the odds of oesophageal cancer patients HRQoL decreased by 3% (AOR = 0.97 95% CI = 0.964–0.995). As there is a unit increase in chocking, the odds of good HRQoL decrease by 2% (AOR = 0.98 95% CI = 0.978–0.998). The odds of good HRQoL increased by 2%, as there was a unit increase in role functioning (AOR = 1.02, 95% CI = 1.01–1.03). The odds of good HRQoL decrease by 2% as there is a unit increase in financial difficulty (AOR = 0.9895% CI = 0.957–0.996) (Table 4).

#### Final model of oesophageal cancer patients

The multicollinearity test and the omnibus test of model coefficients were conducted and the results are presented in Supplemental Table 4.

## Discussion

This study revealed the poor HRQoL experienced by oesophageal cancer patients at TASH in Ethiopia and the components of HRQoL that were most adversely affected. This information can be used to develop interventions to improve the HRQoL of these patients.

Most studies on the HRQoL of oesophageal cancer have focused on assessing how curative treatments, such as surgery and chemoradiation, improve the HRQoL of patients with oesophageal cancer in developed countries, where patients typically present at an early stage [17–20]. Some studies have also assessed the effect of palliative treatments, such as stent placement or oesophageal brachytherapy on HRQoL [21, 22]. In contrast, our study presents a different population of patients from a low-income country, where patients generally present at an advanced stage, have very limited options for curative treatment and lack palliative treatments, such as oesophageal stents or brachytherapy.

The results of this study showed that oesophageal cancer patients at TASH in Addis Ababa, Ethiopia have a reduced HRQoL, as evidenced by the overall mean score of 35.43 on the EORTC QOL C-30 evaluation. Furthermore, the mean scores for the functional scales revealed that cognitive function was the highest, but role function was the lowest, suggesting a greater impact of oesophageal cancer on role functioning than on cognitive functioning. Similarly, the symptom scale results showed the highest score for pain and the lowest score for diarrhoea, indicating a greater presence of pain-related symptoms. Additionally, the mean score for financial difficulties was 90.90, suggesting a substantial financial burden for oesophageal cancer patients. These findings are far lower than those of previous studies that assessed the HRQoL of patients with oesophageal cancer. For instance, a study conducted in China found an overall mean global HRQoL score of 58.33 on the EORTC C-30 evaluation, with the highest score for cognitive function and the lowest for role function [6].

The results also demonstrated that oesophageal cancer patients experienced a range of symptoms, as measured by the EORTC QLQ-OES-18. Eating difficulty was the most reported symptom, followed by dry mouth, trouble with taste, trouble with coughing and trouble with talking. Moderate levels of pain and reflux have been reported. These results are consistent with previous research which has found that patients with oesophageal cancer experience a range of symptoms, including eating difficulty, dry mouth and pain [23].

The results showed that dysphagia, choking, role functioning and financial difficulty significantly affected the HRQoL of patients with oesophageal cancer. For every unit increase in dysphagia, the odds of oesophageal cancer patients experiencing good HRQoL decreased by 3%, whereas the odds of experiencing good HRQoL decreased by 2% for every unit increase in choking. Conversely, the odds of experiencing good HRQoL increased by 2% for every unit increase in role functioning, and decreased by 2% for every unit increase in financial difficulty. Previous studies have indicated that dysphagia, choking, role functioning and financial difficulty are associated with decreased QoL in patients with oesophageal cancer. Dysphagia has been widely reported to have a considerable negative effect on HRQoL in patients with oesophageal cancer [22]. This was demonstrated by comparing the baseline and change after treatment with palliative procedures such as self-expanding stents [22]. Financial difficulties were also found to be significantly associated with HRQoL scores among patients with cancer patients, including those with oesophageal cancer [24]. The results of this study revealed findings that differ from those of other studies. In one study, patients with oesophageal cancer reported physical function, fatigue, and pain as the main factors impacting their HRQoL [25]. Another study identified four symptom clusters associated with HRQoL: psychological, somatic, dysphagia, fatigue-pain and gastrointestinal [26]. The results conducted in China also concluded that the most influential factors for HRQoL in individuals with oesophageal cancer were nutritional status and educational level [6].

Our results showed that physical, role and Cognitive Function scales were significantly associated with HRQoL on bivariate regression analysis, while fatigue, pain, nausea and vomiting, dyspnoea, and financial difficulties were significantly associated with HRQoL among the symptom scales/items only on bivariate logistic regression analysis. Of the factors assessed under QLQ-OES18, only physical function was shown to have a statistically significant association with HRQoL. The results of this study also revealed that the symptom scale of the EORTC QLQ-OES-18 did not show any significant association between HRQoL and emotional function, social function, insomnia, appetite loss, constipation and diarrhoea.

## Limitations

Limitations of this study include the use of self-reported measures, which may be subject to recall bias. Additionally, no pre-and post-treatment HRQoL comparison analyses were conducted, which could have altered the quality of patients’ lives. We did not collect data on the income and educational status of our patients because most of them were farmers and there was no reliable method to measure these variables.

## Conclusion

This study showed that dysphagia, choking, role functioning and financial difficulty are important factors in the HRQoL of patients with oesophageal cancer. Therefore, it is important for healthcare providers to consider these factors when providing care and support to oesophageal cancer patients. We recommend increasing the availability of palliation treatments for dysphagia, including stent and brachytherapy, to improve HRQoL in patients with oesophageal cancer.

## Conflicts of interest

The authors declare no conflicts of interest.

## Funding

This study was funded by Addis Ababa University School of Public Health through the Esophageal Cancer Thematic Research Project award with support from Martin-Luther-University Halle-Wittenberg, Halle (Saale), Germany.

## Ethical approval

Ethical clearance was obtained from the Addis Ababa University Institutional Review Board (IRB) (IRB Reference Number: 096/17/SPH). Written informed consent to participate in this study was obtained from all patients. Confidentiality of the information was maintained throughout the study by excluding names as identification in the data extraction and questionnaire form and the data used only for the purpose of the conducted study.

## Availability of data and materials

The data used for this research are available upon request. The corresponding author will be responsible for supplying materials.

## Author contributions

Jilcha Diribi Feyisa conceptualized the research, was awarded funding for the project, designed the methodology, conducted formal analysis, wrote the original draft and edited subsequent drafts. Mathewos Assefa, Adamu Addissie and Eva Johanna Kantelhardt were responsible for the supervision, methodology and editing. Girum Tesema Zingeta, and Hiwot Seboksa Mideksa contributed to data collection, entry, analysis and editing. Helen Gebre Libanos and Tariku Mengesha involved in analysis.

## Figures and Tables

**Supplemental Table 2.**
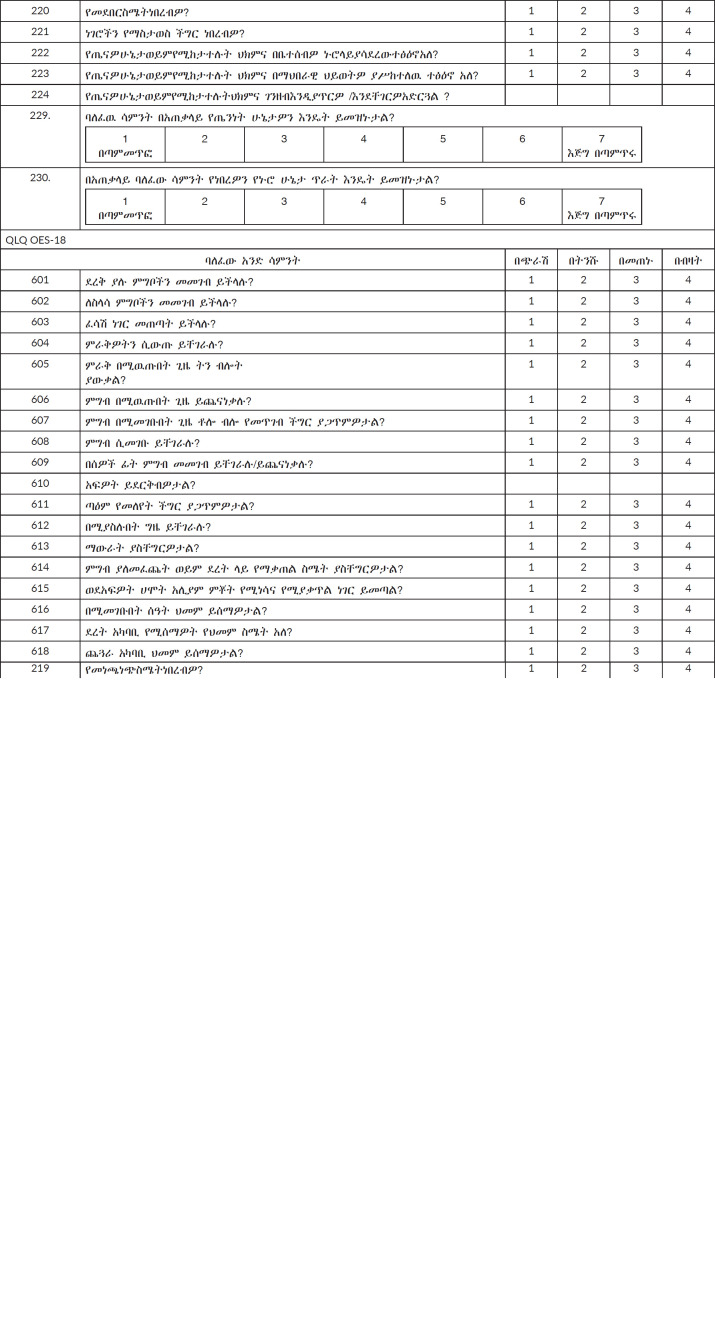
The EORTC QLQ C-30/QLQ OES-18 version three questionnaire Amharic version. Power Geez Unicode1

**Table 1.** Socio-demographic and clinical characteristics of oesophageal cancer patients in TASH, Ethiopia, 2019.

Variable	Category	Frequency	Percentage (%)
Sex	Female	112	56.3
	Male	87	43.7
Marital status	Married	167	83.9
	Single	8	4.0
	Divorced	3	1.5
	Widowed/widower	21	10.6
Occupation	House wife	82	41.2
	Farmer	82	41.2
	Office work	15	7.5
	Other*	20	10.1
Comorbid illness	Hypertension	4	2.0
	Diabetes mellitus	3	1.5
	RVI (Retro viral infection)	6	3.0
Presenting symptom	Dysphagia	198	99.5
	Weight loss	193	97.0
	Heart burn	152	76.4
	Epigastric burning pain	126	63.3
	Vomiting	117	58.8
	Cough	15	7.5
	Hoarseness of voice	1	0.5
Performance status ECOG	0	2	1.0
	I	118	59.3
	II	62	31.2
	III	16	8.0
Clinical staging	I	2	1.0
	II	12	6.0
	III	19	9.5
	IVA	87	43.7
	IVB	70	35.2

* Daily labourers, guard men, house maid, military

**Table 2.** EORTC QLQ C-30/QLQ-OE18 functional and symptom scale scores in oesophageal cancer patients in TASH, Ethiopia, 2019.

EORTC scales (QLQ C-30)	Mean	SD
Global health status/QOL	35.43	18.04
Functional scales		
Physical function	33.90	24.58
Role function	20.79	25.82
Emotion function	46.08	26.50
Cognitive function	62.28	28.25
Social function	27.10	26.82
Symptom’s scales/items		
Fatigue	77.38	24.94
Nausea and vomiting	49.15	33.86
Pain	82.23	20.70
Dyspnoea	50.84	34.52
Insomnia	52.69	36.03
Appetite loss	50.33	40.54
Constipation	60.43	40.16
Diarrhoea	9.76	24.79
Financial difficulties	90.9	16.32
EORTC scales (QLQ-OE18)		
Dysphagia	40.23	24.53
Trouble swallowing saliva	41.75	36.89
Choked when swallowing	39.05	36.18
Eating difficulty	71.04	21.93
Dry mouth	54.71	36.90
Trouble with taste	41.24	35.53
Trouble with coughing	46.12	37.00
Trouble talking	60.60	33.03
Reflux	50.92	29.19
Pain	70.99	23.95

**Table 3.** Functional and symptom scales of EORTC QLQ C-30/QLQ OES18 association with HRQoL among oesophageal cancer in TASH, Ethiopia, 2019.

Variables	COR (95% CI)	*p*-value
EORTC scales (QLQ C-30)		
Functional scales		
Physical function	1.004–1.030	0.009
Role function	1.008–1.033	0.001
Emotional function	0.992–1.015	0.541
Cognitive function	1.002–1.026	0.023
Social function	0.995–1.018	0.243
Symptom scales/items		
Fatigue	0.969–0.993	0.002
Pain	0.966–0.994	0.006
Nausea and vomiting	0.980–0.999	0.025
Dyspnoea	0.976–0.994	0.001
Insomnia	0.983–1.001	0.069
Appetite loss	0.988–1.003	0.266
Constipation	0.988–1.003	0.264
Diarrhoea	0.975–1.005	0.182
Financial difficulties	0.958–0.993	0.006
EORTC scales (QLQ OES18)		
Trouble saliva swallowing	0.988–1.005	0.463
Chocking	0.985–1.003	0.163
Dysphagia	0.974–1.00	0.057
Eating difficulty	0.976–1.004	0.145
Dry mouth	0.986–1.002	0.158
Trouble tasting	0.988–1.005	0.433
Trouble cough	0.982–0.999	0.029
Trouble talking	0.980–0.999	0.029
Reflux	0.987–1.008	0.645
Pain	0.981–1.006	0.310

**Table 4.** Multivariable analysis of variables that have an association with HRQoL among oesophageal cancer in TASH, Ethiopia, 2019.

Variables in the final model	Sig.	Exp(B)	95% CI
Dysphagia	0.013	0.979	0.964–0.995
Chocking	0.025	0.988	0.978–0.998
Role functioning	0.001	1.022	1.01–1.034
Financial difficulty	0.020	0.977	0.957–0.996

**Supplemental Table 1.** The EORTC QLQ C-30/OES-18 version three questionnaire English version.

During the past week		Not at all	A little	Quite a bit	Very much
200.	Do you have trouble doing strenuous activities, like carrying a heavy shopping bag or a suitcase	1	2	3	4
201.	Do you have trouble taking a long walk	1	2	3	4
202.	Do you have trouble taking a short walk outside of the house	1	2	3	4
203.	Do you have need to stay in bed or a chair during the day	1	2	3	4
204.	Do you have need help with eating, dressing, washing yourself or using the toilet	1	2	3	4
During the past week					
205.	Were you limited in doing either your work or other daily activities?	1	2	3	4
206.	Were you limited in pursuing your hobbies or other leisure time activities?	1	2	3	4
207.	Were you short of breath?	1	2	3	4
208.	Have you had pain?	1	2	3	4
209.	Did you need to rest?	1	2	3	4
210.	Have you had trouble sleeping?	1	2	3	4
211.	Have you felt weak?	1	2	3	4
212.	Have you lacked appetite?	1	2	3	4
213.	Have you felt nauseated?	1	2	3	4
214.	Have you vomited?	1	2	3	4
215.	Have you been constipated?	1	2	3	4
During past week					
216.	Have you had diarrhoea?	1	2	3	4
217.	Were you tired?	1	2	3	4
218.	Did pain interfere with your daily activities?	1	2	3	4
219.	Have you had difficulty in concentrating on things, like reading a newspaper or watching television?	1	2	3	4
220.	Did you feel tense?	1	2	3	4
221.	Did you worry?	1	2	3	4
222.	Did you feel irritable?	1	2	3	4
223.	Did you feel depressed?	1	2	3	4
224.	Have you had difficulty remembering things?	1	2	3	4
225.	Has your physical condition or medical treatment interfered with your family life?	1	2	3	4
226.	Has your physical condition or medical treatment interfered with your social activities?	1	2	3	4
228.	Has your physical condition or medical treatment caused you financial difficulties?	1	2	3	4
229.	For the following questions please circle the number between 1 and 7 that best applies to you How would you rate your **overall health** during the past week? 1 Very poor 2 3 4 5 6 7 Excellent				
230.	How would you rate your **overall quality of life** during the past week? 1 Very poor 2 3 4 5 6 7 Excellent				
	EORTC QLQ OES-18				
	**During the past week**	**Not at all**	**A little**	**Quite a bit**	**Very much**
601.	Could you eat solid food?	1	2	3	4
602.	Could you eat liquidised or soft food?	1	2	3	4
603.	Could you drink liquids?	1	2	3	4
604.	Have you had trouble with swallowing your saliva?	1	2	3	4
605.	Have you choked when swallowing	1	2	3	4
606.	Have you had trouble enjoying your meals?	1	2	3	4
607.	Have you felt full up too quickly?	1	2	3	4
608.	Have you had trouble with eating?	1	2	3	4
609.	Have you had trouble with eating in front of other people?	1	2	3	4
610.	Have you had a dry mouth?	1	2	3	4
611.	Have you had problems with your sense of taste?	1	2	3	4
612.	Have you had trouble with coughing?	1	2	3	4
613.	Have you had trouble with talking	1	2	3	4
614.	Have you had acid indigestion or heartburn?	1	2	3	4
615.	Have you had trouble with acid or bile coming into your mouth?	1	2	3	4
616.	Have you had pain when you eat?	1	2	3	4
617.	Have you had pain in your chest?	1	2	3	4
618.	Have you had pain in your stomach?	1	2	3	4

**Supplemental Table 3.** Cronbach alpha value of each item on QLQ OES-18 version three questionnaire Amharic version.

Item	Cronbach's alpha if item deleted
Able to swallow solid foods	0.846
Able to swallow semi solid foods	0.833
Able to drink liquids	0.835
Difficulty of swallowing saliva	0.831
Choking during swallowing	0.827
Discomfort when eating	0.833
Early satiety	0.824
Difficulty in swallowing	0.831
Trouble with eating in front of other people	0.821
Dryness of mouth	0.821
Food and drink taste different from the usual	0.822
Difficulty when cough	0.822
Difficulty in talking	0.824
Indigestion and epigastric pain	0.826
Acid or bile coming to mouth	0.823
Felt pain when eating	0.836
Felt chest pain	0.826
Discomfort in stomach area	0.824

**Supplemental Table 4.** Multicollinearity test among independent variables and omnibus test of model coefficients.

Independent variable	Collinearity diagnostic	
	Tolerance	VIF
Physical function	0.341	2.932
Role function	0.404	2.473
Cognitive function	0.664	1.507
Pain	0.409	2.442
Fatigue	0.268	3.732
Nausea	0.689	1.452
Insomnia	0.730	1.370
Diarrhoea	0.871	1.148
Financial difficulty	0.824	1.214
Choking	0.726	1.378
Dysphagia	0.818	1.222
Eating	0.550	1.817
Dry mouth	0.645	1.550
Trouble coughing	0.566	1.767
Trouble talking	0.506	1.976
Clinical stage at diagnosis	0.869	1.151
Histologic grade	0.884	1.131
VIF-Variance inflation factor		

**Table:** Omnibus test of model coefficients

Variable	Chi-square	Df	Sig.
Esophageal cancer	26.34	4	0.00

DF-Degree of freedom, Sig.-Significance
